# Serum from Hypertensive Patients Induces Cancer-Supporting Phenotype of Vascular Endothelium In Vitro

**DOI:** 10.3390/biom14111374

**Published:** 2024-10-28

**Authors:** Paweł Uruski, Justyna Mikuła-Pietrasik, Andrzej Tykarski, Krzysztof Książek

**Affiliations:** 1 Department of Hypertensiology, Angiology and Internal Medicine, Poznan University of Medical Sciences, Długa 1/2 Str., 61-848 Poznan, Poland; puruski@ump.edu.pl (P.U.); tykarski@o2.pl (A.T.); 2 Department of Pathophysiology of Ageing and Civilization Diseases, Poznan University of Medical Sciences, Święcickiego 4 Str., 60-781 Poznan, Poland; jmikula@ump.edu.pl

**Keywords:** cancer progression, hypertension, vascular endothelium

## Abstract

Background/Objectives: Large-scale epidemiological studies have established a bidirectional association between hypertension and cancer. However, the underlying mechanisms explaining this connection remain unclear. In our study, we investigated whether serum from patients with hypertension (HT) could enhance the aggressiveness of cancer cells in vitro through alterations in endothelial cell phenotype. Methods: Experiments were performed using EAhy926 endothelial cells and ovarian (SKOV-3), colorectal (SW480), pancreatic (PSN-1), breast (MCF-7), and lung (A549) cancer cell lines. Results: This study showed that conditioned medium (CM) produced by EAhy926 cells, when exposed to serum from patients with untreated hypertension (HT-CM), promotes the proliferation, migration, and invasion of every cancer cell line tested. In addition, endothelial cells subjected to HT serum promote the adhesion of all cancer cell types except PSN-1. An intensified transendothelial invasion of cancer cells was accompanied by decreased expression of junctional proteins (connexin 43, E-cadherin, occluding, desmoglein) in HT serum-treated endothelial cells. Quantitative analysis of the secretome of endothelial cells subjected to HT serum showed that they secrete increased amounts of CCL2, CXCL1, CXCL8, bFGF, HGF, IL-6, PAI-1, and TGF-β1. Moreover, cancer cells exposed to HT-CM display increased mRNA expression for several pro-cancerogenic agents, including CXCL8, tPA, and VEGF. Conclusions: Our report shows that hypertension may potentiate cancer cell aggressiveness by modulating endothelial cell phenotype. Further tests with antihypertensive drugs are required to assess whether effective treatment of hypertension can mitigate its cancer-promoting potential.

## 1. Introduction

Primary hypertension (HT) and cancer are chronic and life-threatening diseases commonly associated with modern civilization [1,2]. It is common for these pathologies to occur together: people with hypertension are at risk of developing cancer, and those with cancer may also experience hypertension. This bidirectional relation is not surprising, given their common risk factors (such as obesity, smoking, and diabetes) and underlying mechanisms (like oxidative stress and inflammation) [3]. Strong evidence suggests that hypertension occurs more frequently among cancer patients than in the general population [4]. This association primarily arises from the cellular toxicity and the remodeling of acellular structures caused by radiotherapy and chemotherapy [5]. Several cancer-related symptoms, such as anxiety, pain, and sleeping problems, may continuously elevate blood pressure. Meanwhile, hypertension can also contribute to the development of cancer. Meta-analyses of the literature have revealed a positive association between hypertension and the risk of various cancers, including renal, colorectal, endometrial, prostate, and breast carcinomas [6,7].

Most observations regarding the correlation between hypertension and cancer come from clinical trials, epidemiological studies, and literature meta-analyses [7]. Experimental research focused on the mechanistic aspects of hypertension–cancer interplay is sparse. The cellular and molecular pathomechanisms, especially those underlying the more frequent occurrence of cancer in hypertensive patients, remain somewhat speculative. Several factors may contribute to the increased risk of cancer in patients with hypertension, including changes in the structure and function of the extracellular matrix (ECM) [8,9], increased activity of matrix metalloproteinases (MMPs), particularly MMP-2 and MMP-9 [7], elevated production and activity of vascular endothelial growth factor (VEGF) [10], oxidative stress [11,12], and dysfunction of the renin–angiotensin–aldosterone system (RAAS) [13].

Currently, there is a shortage of in vitro studies demonstrating how hypertension affects the specific phenomena responsible for the aggressiveness of cancer cells. In this study, we investigated whether the vascular endothelium, modified by serum from untreated hypertensive patients, could promote the progression of various cancer cell types. This project is based on the assumption that although hypertension is a disease of large blood vessels (arteries), it modifies serum properties (e.g., its cytokine profile [14]), which may hypothetically translate to the development of unfavorable, cancer-promoting conditions in the whole organism, including the vascular bed. The role of vascular endothelial cells in hypertension is unique. On the one hand, their dysfunction, especially mechanical damage, contributes to the onset of the disease. On the other, they are among the first cells, together with vessel stroma cells, to be affected by hypertension-modified serum [15]. Given the essential role of interactions between endothelial cells and cancer cells in several stages of tumor growth and progression [16,17], it is crucial to examine how HT serum influences these interactions. Understanding this could help explain, at least partly, why patients with hypertension are more predisposed to developing cancer.

## 2. Materials and Methods

### 2.1. Chemicals and Consumables

All chemicals, unless otherwise stated, were purchased from Sigma-Aldrich Corp. (St. Louis, MO, USA). Tissue culture plastics were sourced from Nunc (Roskilde, Denmark).

### 2.2. Hypertensive Patients and Healthy Volunteers

This study analyzed sera from 69 newly diagnosed patients with primary hypertension (HT group; aged 42.4 ± 11.6 years) and 23 healthy volunteers (control group; aged 42.5 ± 12.6 years). According to the 2018 Guidelines issued by the European Society of Cardiology, arterial hypertension is diagnosed when the systolic blood pressure (SBP) is 140 mmHg or higher and/or the diastolic blood pressure (DBP) is 90 mmHg or higher [18]. The exclusion criteria included signs or diagnosis of secondary hypertension, treatment for hypotension, chronic kidney disease with an estimated glomerular filtration rate (eGFR) lower than 60 mL/min/1.73 m^2^, being underage, pregnancy, and breastfeeding.

Medical interviews and examinations were thoroughly conducted for each patient, focusing on identifying potential causes of secondary hypertension and documenting any medications in use. Blood pressure readings in the office were taken with an Omron 705 IT device, calculating the average from three consecutive measurements while the patient was seated. For those diagnosed with hypertension, blood samples were collected during their visit, immediately centrifuged, divided into aliquots, and preserved at −80 °C until analysis was needed. Table 1 presents the basic clinical characteristics of patients with HT compared to control subjects.

### 2.3. Cell Cultures

Ovarian cancer cells (SKOV-3) were obtained from the ECCC (Porton Down, Wiltshire, UK) and grown in RPMI-1640 with L-glutamine (2 mmol/L), penicillin (100 U/mL), streptomycin (100 µg/mL), and 10% fetal bovine serum (FBS). Colorectal cancer cell line SW480 was purchased from the ATCC (Rockville, MD, USA) and maintained in DMEM with L-glutamine (2 mmol/L) and 10% FBS. Pancreatic cancer cell line PSN-1 was purchased from the ECCC and propagated in RPMI-1640 with 10% FBS. Breast cancer cells (MCF7) were obtained from the ATCC and grown in EMEM (ATCC) with 10% FBS and human recombinant insulin (0.01 mg/mL). Lung cancer cells (A549) were obtained from the ATCC and maintained in RPMI 1640 with L-glutamine (2 mmol/L), antibiotics, and 10% FBS. EAhy926 endothelial cells were obtained from the ATCC and cultured in DMEM supplemented with L-glutamine (2 mmol/L), HEPES (25 mmol/L), heparin (10 U/mL), EGF (10 µg/mL), antibiotics, and 20% FBS.

### 2.4. Experimental Protocol

In a study, EAhy926 cell cultures at high density were treated with serum from both HT patients and healthy individuals at a concentration of 20% for 72 h. Following this treatment, the cells were thoroughly rinsed using phosphate-buffered saline (PBS) and then incubated in serum-free conditions for another 72 h to produce a conditioned medium (CM). This protocol was previously established, validated, and detailed elsewhere [19,20]. The CM obtained from the EAhy926 was then applied to various cancer cell lines to assess its impact on several key aspects of cancer progression, including cell proliferation, migration, invasion, the process of epithelial–mesenchymal transition (EMT), and changes in the cancer cells’ gene expression patterns. Additionally, this study explored how cancer cells adhere to the EAhy926 cells following their direct exposure to serum from both HT patients and healthy controls. It also examined the EAhy926 cells for any changes in their function, focusing on the expression levels of proteins that form cell junctions and their secretory profile.

### 2.5. Cancer Cell Progression

The examination of cancer cell proliferation after being exposed for 24 h to CM derived from endothelial cells was conducted using the Cell Proliferation Kit I from PromoKine (Heidelberg, Germany). Furthermore, cancer cells were marked with CellTracker Green BODIPY, a fluorescent marker from Molecular Probes (Eugene, OR, USA). These marked cells were then placed onto a single layer of endothelial cells that had been treated with 20% serum from both HT patients and control donors. This setup was used to measure the direct impact of modified EAhy926 cells on the proliferation of cancer cells. The migration of cancer cells was assessed following a 4 h exposure to the CM from endothelial cells, which served as a chemoattractant, using ChemoTx migration chambers from NeuroProbe (Gaithersburg, MD, USA). The invasion of cancer cells was determined after they were exposed for 24 h to the CM from endothelial cells, utilizing a Cultrex 96-Well BME Cell Invasion Assay from Trevigen Inc. (Gaithersburg, MD, USA), as detailed in [21]. The adhesion of cancer cells, labeled with calcein-AM (Invitrogen, Waltham, MA, USA), to EAhy926 cells that were treated with sera from both HT patients and control donors for 72 h was carried out as described in [22].

### 2.6. Epithelial–Mesenchymal Transition (EMT)

This study observed how cancer cells develop the EMT phenotype after being exposed to endothelial-cell-derived CM for 72 h. This was tracked by examining the levels of vimentin and E-cadherin. The protein expression was analyzed through immunofluorescence, employing antibodies targeting E-cadherin (cat. No. ab15148) and vimentin (cat. No. ab16700), both sourced from Abcam (Cambridge, UK). The concentrations used were 1:100. Additionally, a DyLight 488 antibody (cat. No. ab96899) from Abcam was used at a concentration of 1:500. Rabbit polyclonal IgG (cat. No. ab 37415) and rabbit monoclonal IgG (cat. No. ab172730) purchased from Abcam were used as isotype-matched controls. The fluorescence was recorded using a Synergy^TM^ 2 spectrofluorometer (BioTek Instruments, Winooski, VT, USA) [23].

### 2.7. Intracellular Junctions

This study measured the levels of junctional proteins in EAhy926 cells after exposure to sera from hypertensive patients and healthy controls for 72 h. This was achieved using immunofluorescence and antibodies targeting several proteins: connexin 43 (Abcam, cat. No. ab11370, used at a dilution of 1:100), E-cadherin (Abcam, cat. No. ab15148, also at a 1:100 dilution), occludin (Novus Biologicals, Littleton, CO, USA, cat. No. NBP1-87402, diluted at 1:100), and desmoglein (Abcam, cat. No. ab12077, with a dilution of 1:10), as described in [24]. Rabbit polyclonal IgG (cat. No. ab 37415) and mouse monoclonal IgG (cat. no. ab.170190) obtained from Abcam were used as isotype-matched controls. Fluorescence was quantified using the Synergy^TM^ 2 spectrofluorometer.

### 2.8. Endothelial Cell Secretome

The levels of 10 arbitrarily chosen cytokines, which are secreted into conditioned media by endothelial cells after being exposed for 72 h to serum from patients with hypertension and healthy control volunteers, were measured using specific DuoSet^®^ Immunoassay Development kits (R&D Systems, Minneapolis, MN, USA), according to the manufacturer’s instructions.

### 2.9. Cancer Cell Transcriptome

Cancer cells were treated with endothelial-cell-derived conditioned medium for a period of 72 h. Following this treatment, the levels of mRNA for nine genes linked to the progression of cancer were examined using quantitative PCR (qPCR). To carry out this analysis, total RNA was extracted using TRI Reagent^®^ (Merck, Darmstadt, Germany) and purified with Clean-up RNA Concentrator kit (A&A Biotechnology, Gdynia, Poland). The purity of the RNA was confirmed using the A260/A280 ratio, which was within 1.9–2.0. To begin with, 2 μg of RNA underwent reverse transcription using the GoScript™ Reverse Transcription System by Promega Corporation (Madison, WI, USA). The qPCR was executed with the help of PowerUp™ SYBR™ Green Master Mix provided by Thermo Fisher Scientific (Waltham, MA, USA), alongside suitable primer pairs sourced from OriGene (Rockville, MD, USA). These procedures were all conducted using a LightCycler^®^ 96 System from Roche (Basel, Switzerland). The operational conditions were set as follows: an initial 2 min at 50 °C, followed by 2 min at 95 °C, and then 45 cycles consisting of 15 s at 95 °C and 1 min at 60 °C. To assess the quality of each generated amplicon, the dissociation curves were analyzed at the PCR’s Melt Curve Stage. For accuracy, each reaction plate included a negative control and an internal control. The relative expression levels of the genes were calculated using the 2^−ΔΔCt^ method, utilizing the glyceraldehyde-3-phosphate dehydrogenase (hGAPDH) gene as a reference. The primer sequences applied in the RT-qPCR are detailed in Table 2.

### 2.10. Statistics

Statistical analysis was performed using GraphPad Prism™ v.6.00 software (GraphPad Software, San Diego, CA, USA). The means were compared using the Mann–Whitney test. The results are expressed as the mean ± S.D. Differences with a *p*-value < 0.05 were considered to be statistically significant.

## 3. Results

### 3.1. Serum from HT Patients Promotes Endothelial-Cell-Dependent Cancer Cell Progression In Vitro

**Figure 1:**
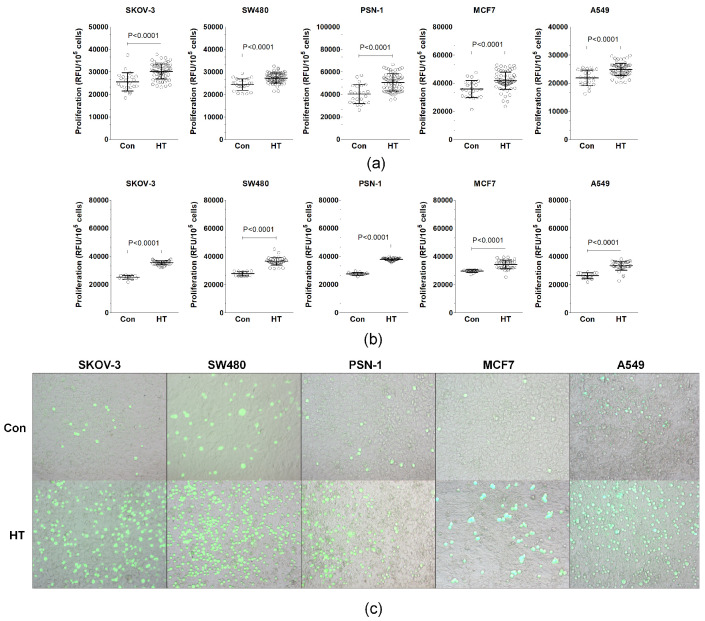
Effect of serum from hypertensive (HT) patients and control donors (Con) on the proliferation of cancer cells dependent on endothelial cells. The effect of endothelial-cell-derived conditioned medium (CM) (**a**) and the direct contact between serum-exposed endothelial cells and cancer cells (**b**) on cancer cell proliferation. Representative images of cancer cells probed with CellTracker Green (green) proliferating atop EAhy926 endothelial cells (**c**). The experiments utilized sera from 23 healthy donors and 69 hypertensive patients. Scatter dot plots include mean ± S.D. values. RFU stands for relative fluorescence units. Magnification × 20.

EAhy926 vascular endothelial cells were treated with 20% serum from HT patients and healthy controls. The conditioned medium (CM) produced by these cells, labeled HT-CM for those from the HT patient serum and Con-CM for those from the control serum, was then used to treat various cancer cell lines to assess their progression-related behaviors. This study found that HT-CM consistently enhanced the growth of the SKOV-3, SW480, PSN-1, MCF7, and A549 cancer cell lines more than Con-CM did, as shown in Figure 1a. This effect was more pronounced when cancer cells were directly grown on EAhy926 cells that had been pre-treated with the sera under study, as illustrated in Figure 1b,c.

**Figure 2:**
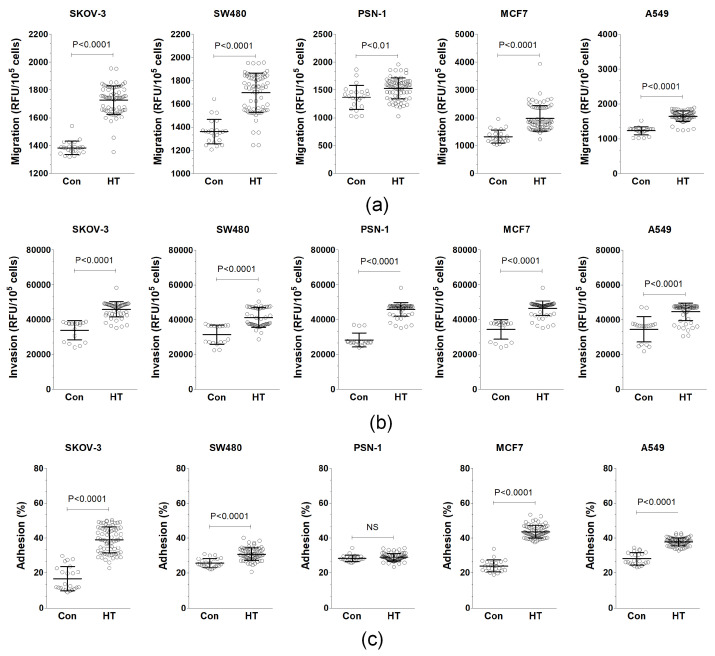
The impact of serum from hypertensive (HT) patients and control donors (Con) on the endothelial-cell-dependent progression of cancer cells. The effect of endothelial-cell-derived conditioned media (CM) on cancer cell migration (**a**) and transendothelial invasion (**b**). The adhesion of cancer cells to EAhy926 endothelial cells when exposed to HT and control sera (**c**). These experiments utilized sera from 23 healthy donors and 69 hypertensive patients. Scatter dot plots include mean ± S.D. values. RFU stands for relative fluorescence units. N.S. indicates a lack of significant difference.

This study found that HT-CM and Con-CM both acted as attractants for cancer cells, improving their migration and invasion. When compared to Con-CM, HT-CM was more effective in prompting all tested cancer cell lines to migrate, as illustrated in Figure 2a. Furthermore, in experiments measuring the ability of cancer cells to penetrate through endothelial cells layered over a basement membrane extract (BME), HT-CM’s stimulating impact was significantly greater than that of Con-CM for every cancer cell line examined, shown in Figure 2b. Additionally, when EAhy926 cells, which had been treated with either HT or control sera, were used as a surface, all cancer cell lines, with the exception of PSN-1, showed improved adherence to the endothelial cells that had interacted with HT-CM, as documented in Figure 2c.

### 3.2. Serum from HT Patients Affects EMT Phenotype and Expression of Junctional Proteins

**Figure 3:**
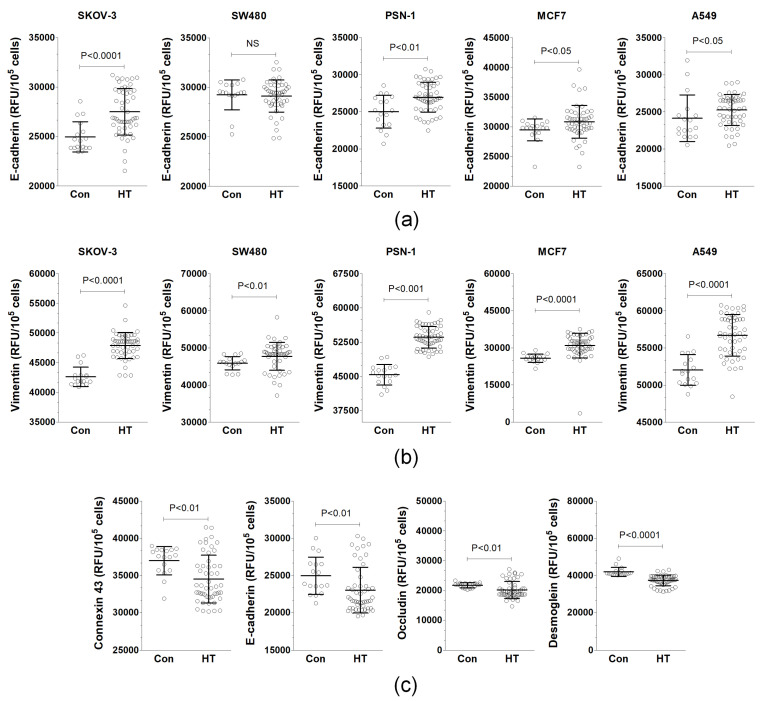
Plausible mechanisms through which serum from hypertensive (HT) patients increases the invasion of cancer cells dependent on endothelial cells. Effect of endothelial-cell-derived conditioned medium (CM) on the development of epithelial–mesenchymal transition (EMT) in cancer cells, as indicated by the expression of E-cadherin (**a**) and vimentin (**b**). The impact of serum from HT patients and control donors on the expression of junctional proteins in EAhy926 cells (**c**). The experiments were conducted using sera from 17 healthy donors and 51 hypertensive patients. Scatter dot plots include mean ± S.D. values. RFU stands for relative fluorescence units. N.S. denotes not significant.

This study explored how cancer cells develop the EMT (epithelial–mesenchymal transition) phenotype when exposed to HT-CM (endothelial-cell-derived conditioned medium) and Con-CM (control conditioned medium). This was carried out by observing changes in the levels of E-cadherin, a marker of epithelial cells, and vimentin, a marker of mesenchymal cells. Surprisingly, when exposed to HT-CM, nearly all cancer cell lines showed an increase in E-cadherin levels, except for one (SW480), as shown in Figure 3a. In terms of vimentin, exposure to HT-CM led to increased expression in every cancer cell line tested, in comparison to those treated with Con-CM, as illustrated in Figure 3b.

This study also investigated the expression of four junctional proteins—connexin 43, E-cadherin, occludin, and desmoglein—in the EAhy926 cells exposed to serum from hypertensive (HT) patients and compared it to cells treated with serum from healthy individuals. This was carried out to determine if changes in the levels of these proteins could contribute to the observed increase in cancer cell invasion across endothelial barriers seen with HT patient serum. The findings revealed that serum from HT patients significantly decreased the expression of all four proteins studied in comparison to the expression levels in cells treated with serum from healthy volunteers (Figure 3c).

### 3.3. Serum from HT Patients Stimulates the Production of Pro-Cancerogenic Cytokines by Endothelial Cells

**Figure 4:**
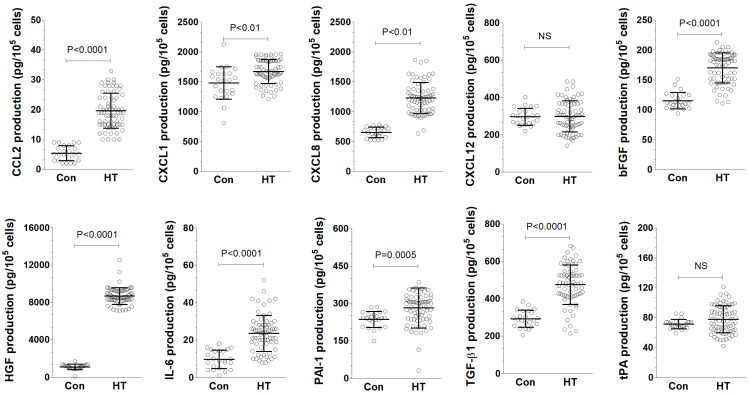
The effect of serum from hypertensive patients (HT) and control donors (Con) on the production of cancer-promoting cytokines in CM by EAhy926 was investigated. The experiments utilized sera from 23 healthy donors and 69 hypertensive patients. Scatter dot plots, including mean ± S.D. values, were presented. N.S. indicates not significant.

This study measured the secretion levels of certain proteins by EAhy926 cells after they were treated with HT and control sera using an ELISA method. The findings revealed that the conditioned medium (CM) from endothelial cells treated with HT serum (HT-CM) had higher concentrations of several molecules—CCL2, CXCL1, CXCL8, bFGF, HGF, IL-6, PAI-1, and TGF-β1. However, the levels of CXCL12 and tPA remained the same when compared to the CM from the control group (Con-CM), as illustrated in Figure 4.

### 3.4. Serum from HT Patients Modulates Endothelial-Cell-Dependent Expression of Growth-Promoting TRANSCRIPTS in Cancer Cells

Cancer cells were exposed to endothelial-cell-derived HT-CM and Con-CM to establish differences in the expression of mRNA for 10 arbitrarily selected targets involved in cancer growth and metastasis. The results of RT-qPCR analysis showed that HT-CM increased the expression of several of the tested genes, and this effect varied depending on the cell type. Specifically, in SKOV-3 cells, HT-CM up-regulated the levels of mRNA for CXCL1, CXCL8, FGF5, IL-6, tPA, and VEGF. In SW480 cells, HT-CM boosted mRNA levels for TGF-β1, tPA, and VEGF. PSN-1 cells experienced an increase in mRNA for all genes studied, except for FGF5, upon exposure to HT-CM. In MCF7 cells, HT-CM increased mRNA levels for CCL2, CXCL1, CXCL8, TGF-β1, tPA, and VEGF. Another cell type (A549) exhibited the most significant response to HT-CM, with increased mRNA levels for all tested genes except FGF5, which was undetectable. It was also observed that the mRNA for CXCL8, tPA, and VEGF was consistently overexpressed in response to HT-CM across all the cancer cell lines tested, as shown in Table 3.

## 4. Discussion

Reciprocal interactions between endothelial cells and cancer cells play an important role at different stages of cancer development. Efficient angiogenesis is critical for the growth of both primary tumors and their metastases [16]. Additionally, endothelial cells continuously interact with cancer cells via soluble agents and direct contact during intra- and extravasation processes, playing a role in cancer spread to distal locations [17]. While large epidemiological studies have shown a link between hypertension and cancer [6,7], experimental research directly proving a cause-and-effect relationship between these two pathologies is limited.

In our study, we examined the possibility that serum from patients with primary hypertension (HT), known for its changed biochemical composition, including levels of cytokines involved in different stages of cancer development, might have the potential to promote cancer [14]. Our research has demonstrated that using a 20% concentration of serum from HT patients in vitro can change the characteristics of endothelial cells to support cancer development in just 72 h. Previously, the analogical experimental setup was effective in showing that serum from patients with varicose veins exhibits characteristics that promote senescence and inflammation [19,20]. It can, therefore, be assumed that prolonged and/or continuous exposure of the vascular endothelium in vivo could lead to more severe clinical effects.

A key aspect of how HT serum supports cancer growth is its impact on endothelial cells. Essentially, when endothelial cells are influenced by HT serum, they produce a changed set of secreted factors. This altered secretome from the endothelial cells stimulates various cancer cells to proliferate, migrate, and invade.

From a detailed perspective, the increased motility of cancer cells is probably linked to the activity of particular proteins that are excessively secreted into the surrounding environment by endothelial cells. This process enhances interactions with specific receptors on the surface of cancer cells, which then triggers the activation of certain signaling cascades. The behavior of cancer cells may have been further enhanced due to the formation of a self-stimulating cycle. In this cycle, the HT-CM increases the expression of specific transcripts coding for proteins responsible for aggressive cancer cell behavior. The proteins that are overly produced by endothelial cells or overly expressed by cancer cells include many well-known factors that promote cancer cell progression. These factors are CCL2, CXCL1, CXCL8, bFGF, FGF5, HGF, IL-6, PAI-1, TGF-β1, and VEGF.

Current research indicates that the proteins mentioned may play a role in hastening the progression of a very broad spectrum of cancers. However, our research concentrated on five arbitrarily selected cancer types; so, it is crucial to underscore those results that are especially consistent with our experimental frameworks. As per chemokines, CCL2 stimulates the proliferation of ovarian cancer cells via the MAPK/ERK pathway [25]. This chemokine has a similar impact on lung cancer cells [26]. CXCL1 supports the proliferation of colorectal cancer cells by activating the NF-κB/P300 signaling pathway [27]. In the same type of cancer, CXCL8 mediates the intensification of aerobic glycolysis and restricts the production of reactive oxygen species (ROS), which subsequently translates to enhanced cell proliferation and invasion [28]. For the remaining agents, HGF promotes the proliferation of ovarian cancer cells by up-regulating c-Met/PI3K/Akt and GRP78 signaling [29]. IL-6, in turn, supports pancreatic cancer cell proliferation by down-regulating miR-455-5p [30]. FGF5 is known to promote the replication, migration, and invasion of non-small-cell lung cancer cells [31]. Another type of fibroblast growth factor, specifically bFGF, in turn, promotes the growth of cancer cells through the Ras pathway. Its activation triggers a series of Ser/Thr Raf kinases, as well as MEK and ERK1/2 [32,33]. Even VEGF, classically considered a key angiogenic agent, is known to promote the mitogenic activity of breast cancer cells utilizing MAPK/ERK signaling [34]. Additionally, ovarian cancer cells have increased growth when treated with an exogenous, recombinant form of VEGF [35].

A gradient created by chemokines that are released in excess into the environment (HT-CM) can also play a role in enhancing the migration of cancer cells. For instance, CCL2 mediates the migratory response of breast [36] and lung cancer cells [26]. In addition, CXCL1 and CXCL8 promote the migration of colorectal cancer cells [27,37]. CXCL8 also plays a role in aiding this process within ovarian cancer cells [38]. Interestingly, it is also part of a group of proteins that contribute to the enhanced migration of ovarian cancer cells when subjected to senescent peritoneal mesothelium [35]. Non-chemokine agents, such as HGF, stimulate the migration of ovarian cancer cells [39], while IL-6 supports this process in pancreatic cancer cells [40]. The list of pro-migratory proteins hypersecreted by endothelial cells in response to HT serum or transcripts up-regulated in cancer cells exposed to endothelial-cell-derived HT-CM also includes bFGF [41], FGF5 [31], and TGF-β1 [42].

Cancer cells show a higher rate of invading through the endothelium when exposed to conditioned media from endothelial cells treated with serum from HT patients. This invasion process includes both the physical penetration of the endothelial barrier and the chemical breakdown of the nearby supportive tissue [43]. Additionally, the EMT, a unique morphological transformation, further supports these processes by enhancing the motility of cancer cells [44]. In our experimental model, we found that the endothelial HT-CM was rich in various agents known to trigger EMT, such as TGF-β1 [45], CXCL1 [46], and HGF [47]. Notably, TGF-β1 and CXCL1’s mRNA levels were significantly higher in cancer cells treated with HT-CM. However, the transition of these cancer cells to a full EMT phenotype was not completed. This was evidenced by the increased production of the mesenchymal marker, vimentin, which did not coincide with a decrease in the epithelial marker, E-cadherin. Surprisingly, the level of E-cadherin was also found to be elevated. In this context, one may speculate that HT-CM may elicit dynamic transitioning between epithelial and mesenchymal states. This phenotypic plasticity, or simply the partial EMT, may suggest that cancer cells have become more adaptable to different conditions and specific needs. This aligns with modern perspectives on EMT, suggesting that cancer cells often experience a partial EMT, adopting mesenchymal characteristics while still maintaining epithelial markers, notably E-cadherin [48,49]. For example, the traits of mesenchymal cells could, in some ways, enhance their ability to move and invade, while epithelial traits might support their collective migration and organized invasion [50,51].

Another probable reason for the intensified transendothelial invasion is the decreased expression of intracellular junctions within endothelial cells treated with HT serum, which increases the endothelial barrier’s permeability to cancer cells. The correlation between the reduced expression of connexin 43, E-cadherin, occludin, and desmoglein in normal cells (the peritoneal mesothelium) and the increased invasiveness of cancer cells has been previously demonstrated in various ovarian cancer cell lines [21]. VEGF, which is overexpressed by cancer cells treated with HT-CM, may also play a role in this process. As observed in breast cancer cells, VEGF supports their transendothelial passage by impairing junctional proteins within endothelial cells, specifically by interfering with the VE-cadherin–β-catenin complex [52]. Furthermore, the invasive properties of cancer cells are likely intensified by PAI-1 and tPA, which are elevated in HT-CM and overexpressed by cancer cells treated with HT-CM. The ability of PAI-1 and tPA to modulate the cellular cytoskeleton and reorganize the stromal compartment, thereby facilitating cancer cell movement, has been repeatedly described [53,54]. Finally, some chemokines hypersecreted to endothelium-derived HT-CM may also fuel cancer cell invasion. This may be the case, e.g., for CXCL8 and CXCL12, which have previously been found to cooperate in stimulating the invasion and proliferation of pancreatic cancer cells [55].

## 5. Conclusions

Collectively, our report proves experimentally that serum from HT patients potentiates the pro-cancerogenic capacity of vascular endothelium in vitro. Intensified endothelial–cancer cell crosstalk may likely contribute to a significant degree to an exacerbation of cancer in hypertensive patients. Further research using various classes of antihypertensive drugs is urgent to establish whether effective HT treatment will modulate the pro-cancerogenic phenotype of endothelial cells and thus counteract hypertension-related tumor development. This postulate gains strength from the fact that existing data on this topic are inconclusive. For instance, a detailed investigation revealed that angiotensin receptor blockers might slightly elevate the risk of cancer. Conversely, other research indicates that using these drugs can decrease cancer risk. Similarly, another study showed lower cancer risk in patients treated with beta blockers. Moreover, a comprehensive review of 27 randomized controlled trials covering 56 different treatment groups found no link between antihypertensive medications and cancer [56,57,58,59].

## Figures and Tables

**Table 1:** Characteristics of patients diagnosed with hypertension and the control group individuals who provided serum samples.

Parameter	Control Donors	HT Patients
Number of patients (N),	23	69
Sex (Male/Female)	14/9	50/19
Age (years)	42.5 ± 12.6	42.4 ± 11.6
Office SBP (mmHg)	116.4 ± 27.1	145.5 ± 14.5
Office DBP (mmHg)	69.6 ± 16.6	87.7 ± 9.8

SBP—systolic blood pressure; DBP—diastolic blood pressure. The results are expressed as mean ± SD.

**Table 2:** The sequences of primers utilized in real-time qPCR reactions.

Gene	Forward	Reverse
*CCL2*	AGAATCACCAGCAGCAAGTGTCC	TCCTGAACCCACTTCTGCTTGG
*CXCL1*	AGCTTGCCTCAATCCTGCATCC	TCCTTCAGGAACAGCCACCAGT
*CXCL8*	GAGAGTGATTGAGAGTGGACCAC	CACAACCCTCTGCACCCAGTTT
*IL-6*	AGACAGCCACTCACCTCTTCAG	TTCTGCCAGTGCCTCTTTGCTG
*tPA*	TGGTGCTACGTCTTTAAGGCGG	GCTGACCCATTCCCAAAGTAGC
*PAI-1*	CTCATCAGCCACTGGAAAGGCA	GACTCGTGAAGTCAGCCTGAAAC
*FGF5*	GGAATACGAGGAGTTTTCAGCAAC	CTCCCTGAACTTGCAGTCATCTG
*TGF-β1*	TACCTGAACCCGTGTTGCTCTC	GTTGCTGAGGTATCGCCAGGAA
*VEGF*	TTGCCTTGCTGCTCTACCTCCA	GATGGCAGTAGCTGCGCTGATA
*GAPDH*	GTCTCCTCTGACTTCAACAGCG	ACCACCCTGTTGCTGTAGCCAA

**Table 3:** Effect of serum from hypertensive patients (HT) and control donors (Con) on the endothelial-cell-dependent expression of cancer-promoting transcripts in SKOV-3, SW480, PSN-1, MCF7, and A549 cells.

Gene	SKOV-3		SW480		PSN-1	
	Con	HT	Con	HT	Con	HT
*CCL2*	n.d.	n.d.	1.1 ± 0.4	1.5 ± 0.7	1.0 ± 0.1	1.7 ± 0.8 **
*CXCL1*	1.0 ± 0.2	3.4 ± 1.7 ***	1.1 ± 0.4	2.9 ± 3.7	1.0 ± 0.2	0.4 ± 0.2 **
*CXCL8*	1.0 ± 0.2	4.3 ± 1.8 ***	n.d.	n.d.	1.0 ± 0.1	1.5 ± 1.3 ***
*FGF5*	1.0 ± 0.2	3.2 ± 1.7 **	1.0 ± 0.1	1.3 ± 0.6	1.0 ± 0.1	0.9 ± 0.2
*IL-6*	1.0 ± 0.1	3.8 ± 1.7 ***	1.0 ± 0.3	5.2 ± 7.4	1.0 ± 0.2	0.8 ± 0.4 **
*PAI-1*	n.d.	n.d.	1.0 ± 0.2	12.7 ± 19.3	1.0 ± 0.2	0.6 ± 0.1 **
*TGFβ1*	1.0 ± 0.1	1.1 ± 0.3	1.1 ± 0.1	1.3 ± 0.2 ***	1.0 ± 0.1	1.3 ± 0.3 ***
*tPA*	1.0 ± 0.3	16.9 ± 10.4 ***	1.0 ± 0.2	6.5 ± 4.6 ***	1.0 ± 0.1	0.5 ± 0.2 ***
*VEGF*	1.0 ± 0.1	2.5 ± 1.0 ***	0.9 ± 0.3	3.0 ± 1.0 ***	1.0 ± 0.2	1.7 ± 0.4 ***
**Gene**	**MCF7**		**A549**			
	**Con**	**HT**	**Con**	**HT**		
*CCL2*	1.0 ± 0.3	2.8 ± 1.6 **	1.0 ± 0.2	7.0 ± 2.1 ***		
*CXCL1*	1.0 ± 0.3	11.5 ± 3.8 ***	1.0 ± 0.1	4.4 ± 2.5 ***		
*CXCL8*	1.0 ± 0.1	10.4 ± 2.5 ***	1.0 ± 0.1	8.8 ± 3.4 ***		
*FGF5*	n.d.	n.d.	n.d.	n.d.		
*IL-6*	n.d.	n.d.	1.0 ± 0.1	50.6 ± 32.2 **		
*PAI-1*	n.d.	n.d.	1.0 ± 0.1	13.2 ± 2.9 ***		
*TGFβ1*	1.0 ± 0.1	3.6 ± 0.9 ***	1.0 ± 0.1	1.3 ± 0.3 **		
*tPA*	1.0 ± 0.2	6.2 ± 2.0 ***	1.0 ± 0.1	2.0 ± 0.4 ***		
*VEGF*	1.0 ± 0.1	1.7 ± 0.4 ***	1.0 ± 0.2	7.6 ± 2.2 ***		

In this study, cancer cells were treated with conditioned media derived from endothelial cells, specifically HT-CM and Con-CM, for 72 h. This experiment utilized sera collected from 8 control donors and 24 HT patients. qPCR tests were conducted in duplicate to ensure accuracy. The data are presented as mean ± S.D. values. A significance level of *p* < 0.01 is denoted by “**”, and a level of *p* < 0.001 is indicated by “***”, both compared to the control (Con). “n.d.” signifies that the result was not detected.

## Data Availability

The original contributions presented in the study are included in the article, further inquiries can be directed to the corresponding author.
